# Role of mast cells in the pathogenesis of oral submucous fibrosis: a systematic review and meta-analysis

**DOI:** 10.1186/s12903-024-05025-8

**Published:** 2024-10-14

**Authors:** Laliytha Kumar Bijai, Ali A. Aboalela, Farraj Albalawi, Sanjeev B Khanagar, Kiran Iyer

**Affiliations:** 1 Maxillofacial Surgery and Diagnostic Sciences Department, College of Dentistry, King Saud bin Abdulaziz University for Health Sciences, Riyadh, 11426 Saudi Arabia; 2 King Abdullah International Medical Research Centre, Ministry of National Guard Health Affairs, Riyadh, 11481 Saudi Arabia; 3 Preventive Dental Science Department, College of Dentistry, King Saud bin Abdulaziz University for Health Sciences, Riyadh, 11426 Saudi Arabia

**Keywords:** Mast cell count, Intact mast cell, Degranulated mast cell, Oral submucous fibrosis, Toluidine blue, Immunohistochemistry

## Abstract

**Background:**

Oral Submucous Fibrosis (OSMF) is an oral potentially malignant disorder (OPMD) that commonly occurs in the South Asian population as there is high usage of areca nut. There has been extensive research on the pathogenesis and treatment of this condition. It is well-established in the scientific literature that mast cells (MC) have a definitive role in several inflammatory disorders. OSMF being a chronic inflammatory disorder, is also expected to have increased MCs. Hence, this review aims to evaluate the role of MCs in the pathogenesis of OSMF.

**Methods:**

A systematic search of articles was performed by two of the authors independently in PubMed, Scopus, Embase, Web of Science, and Google Scholar using the appropriate keywords and Boolean terms. The risk of bias was assessed using the Modified Newcastle-Ottawa Scale. The meta-analysis was performed with R studio software (Version: 4.4.0, Year: 2024, Company: The R foundation for statistical computing).

**Results:**

The search retrieved 36 studies, of which 16 were suitable for the review. There is evidence for a marked increase in the number of MCs in OSMF than the normal mucosa upon analyzing the retrieved articles. However, when comparing the grades of OSMF, there are variations in the reports. As all the retrieved articles were case-control studies, the risk of bias was analyzed using the Modified New Castle Ottawa Scale. All the studies scored in the good category (Score 6–9). The pooled effect size shows the Standard Mean Deviation (SMD) to be 0.09, 95% confidence interval (CI) [-0.18;0.37] to lie on either side of no effect. Hence the role of MCs in OSMF has not been established because of homogeneity and consistent sampling error.

**Conclusion:**

Our systematic review does suggest a definitive role of mast cells in the progression of OSMF. However, there is a lack of methodological rigor in the included studies. This may contribute to diluting the results.

## Background

Atrophia idiopathic mucosae oris was the name originally given for OSMF by Schwartz J based on observations of five Indian females residing in the Kenyan (Africa) region [1]. The prevalence of OSMF is around 0.1–30% predominantly observed in the South Asian Region, namely in India, Pakistan, and Sri Lanka [2, 3]. The major etiological factor reported in the literature for OSMF is the areca alkaloids derived from areca nuts. The use of areca nuts individually or in combination with lime slake on a beetle leaf, stems from the fact that they are deeply embedded in the culture of the geographic region [4].

The most common and earliest clinical complaint of patients with OSMF is a burning sensation aggravated by consuming spicy foods. There are also features of erythema, a marble-like appearance of oral mucosa. OSMF patients also can exhibit vesicles, that rupture to form ulcers which in turn heal by fibrosis that slowly leads to restricted mouth opening. Loss of hearing, dysphagia, restricted tongue movement, difficulty in speech, and mastication are some of the other features of OSMF [2]. There are several classification systems in the literature for OSMF. However, the most widely used classification of OSMF was given by Pindborg JJ and Sirsat SM as very early, early, moderately advanced, and advanced stages [5]. Histopathological changes observed and discussed in the literature point towards complex by-products that initiate growth factors, TNF-α, and decreased matrix metalloproteinases (MMP) which eventually lead to fibroblast proliferation beyond normal and a decrease in collagen breakdown [6].

Sir Paul Ehrlich in 1877 was the first to identify the mast cells as a granular cell of loose connective tissue [7]. The MCs consist of mediators (histamine, serotonin, kallikrein, TNF-α, tryptase and chymase) stored in the cytoplasmic granules. These mediators are released upon degranulation due to local or systemic conditions [8]. Literature suggests that MCs play a vital role in various physiologic conditions, acute to chronic inflammation of oral mucosa and pathologic conditions including cancer [9, 10]. There is published evidence that the precursor to fibroblast proliferation is the initiation of inflammatory mediators from the MCs [11].

Also, studies suggest that MCs can cause a burning sensation in OSMF due to the histamine release following degranulation. MCs also contribute to fibrosis of the stroma which may lead to malignant changes in the OSMF [6].

However, there is still a debate on the extent of MC involvement in the progression of OSMF, non-homogeneity in procedures pertinent to estimation, staining technique, histopathological classification, inappropriate controls and the ratio of controls enrolled. Multiple factors have consistently produced variations in observations and reporting.

Hence the present review aims to evaluate the role of MCs in the pathogenies of OSMF.

The primary objective of this systematic review was to analyze the MC density and/or count in various grades of OSMF as against the controls (normal mucosa/ OPMD/ Oral squamous cell carcinoma). The secondary objective was to assess the variation in methodology adopted by studies to determine mast cells.

This review attempts to answer the following research questions:

- “Is there an increase in MC expression specifically typical, atypical, granulated, intact, degranulated, and total MCs in OSMF? ”
- “Is there a significant difference in MC expression between various grades of OSMF patients and normal healthy individuals? ”
- “Is there any significant difference in MC expression between OSMF patients and other OPMDs or Oral Squamous Cell Carcinoma (OSCC)? ”
- “Does the staining methods, and counting techniques of MCs influence the outcomes?”

## Materials and methods

**Ethical Concern**: This review was done following the PRISMA guidelines [12] and was approved by the Ethics Committee in King Abdullah International Medical Centre, Riyadh, Saudi Arabia (IRB No: NRC22R/619/11). The review has been registered in PROSPERO (CRD42022378733).

### Eligibility criteria

**Inclusion criteria**: The literature was screened to identify articles satisfying at least 1 of the following requirements: (a)Case-Control studies (b) Retrospective Cohort studies.

#### Exclusion criteria

The articles with the following criteria were excluded from the study (a) Studies other than English (b) in Vitro studies (c) Animal studies (d) Case reports and case series (e) Narrative review (f) Randomised Control Trial.

#### Information sources and search strategy

We did an extensive literature search in PubMed, Scopus, Embase, Web of Science, and Google Scholar with the keywords mentioned for the articles published from the first article published on the role of mast cells in OSMF in 1967 until 2024. The article was extracted based on a one-month search strategy (January- February 2024).

### Selection process

The systematic review was initiated by constructing the PICO: (a) Population: “oral submucous fibrosis”; (b) Intervention: “histopathological staining for MCs”; (c) Control: “normal healthy individuals” “Oral Potentially Malignant Disorder” “Oral Squamous Cell Carcinoma” and (d) Outcome: “qualitative and quantitative assessment of MCs.” The PICO details of the selected studies and the study characteristics are given in Table 1.

The search was conducted using the following keywords and boolean operators. The keywords used were “oral submucous fibrosis,” OR “OSMF” OR “submucous fibrosis” AND “histopathological staining” OR “Toluidine blue” OR “immunohistochemistry” OR “biopsy” AND “Normal mucosa” OR “Oral Potentially Malignant Disorders” OR “Oral Squamous Cell Carcinoma” AND “mast cells,” OR “intact mast cells,” OR “degranulated mast cells,” OR “mast cell count” OR “mast cell density”.

(((((oral submucous fibrosis) OR (OSMF)) OR (Submucous Fibrosis) AND ((ffrft[Filter]) AND (excludepreprints[Filter]) AND (fft[Filter]) AND (english[Filter]))) AND ((((histopathological staining) OR (toluidine blue)) OR (immunohistochemistry)) OR (biopsy) AND ((ffrft[Filter]) AND (excludepreprints[Filter]) AND (fft[Filter]) AND (english[Filter])))) AND (((((normal mucosa) OR (oral potentially malignant disorders)) OR (OPMD)) OR (oral squamous cell carcinoma)) OR (OSCC) AND ((ffrft[Filter]) AND (excludepreprints[Filter]) AND (fft[Filter]) AND (english[Filter])))) AND (((((mast cell) OR (intact mast cell)) OR (degranulated mast cell)) OR (mast cell count)) OR (mast cell density) AND ((ffrft[Filter]) AND (excludepreprints[Filter]) AND (fft[Filter]) AND (english[Filter]))) Filters: Free full text, Full text, English, Exclude preprints, Free full text, Full text, English, Exclude preprints.

The search retrieved a total of 16 articles, which included 12 articles from PUBMED, 8 from Scopus, 2 from EMBASE, 5 from Web of Science, and 9 from Google Scholar.

Relevant articles were analyzed from each of the databases.

### Data collection process

Two independent researchers, Laliytha Kumar Bijai (LKB) and Kiran Iyer (KI), performed data extraction in a Microsoft Excel spreadsheet based on the studies that met the inclusion criteria. Kappa Statistics was used to initially observe the agreement between the two authors. Discrepancy in the inclusion of the study was sorted by discussion. All the PICO-based and other variable-based information was entered into a predesigned Microsoft Excel form by Farraj Albalawi (FA). The variables included were age, gender, classification of OSMF, MC staining technique, MC counting technique, and parameters observed (MC count, MC density, intact MC, degranulated MC, MC tryptase, MC Chymase).

### Study risk of bias assessment

In the final analysis, all articles were observed to be Case-Control Studies. Hence, the authors agreed to utilize the Modified New Castle Ottawa Scale [13].

### Effect measures

The mean mast cell counts and /or density were ascertained from each of the articles included in the study. In the meta-analysis, a random effect model was used and a standard mean difference was observed between the case and control.

## Results

### Study selection

**Fig. 1:**
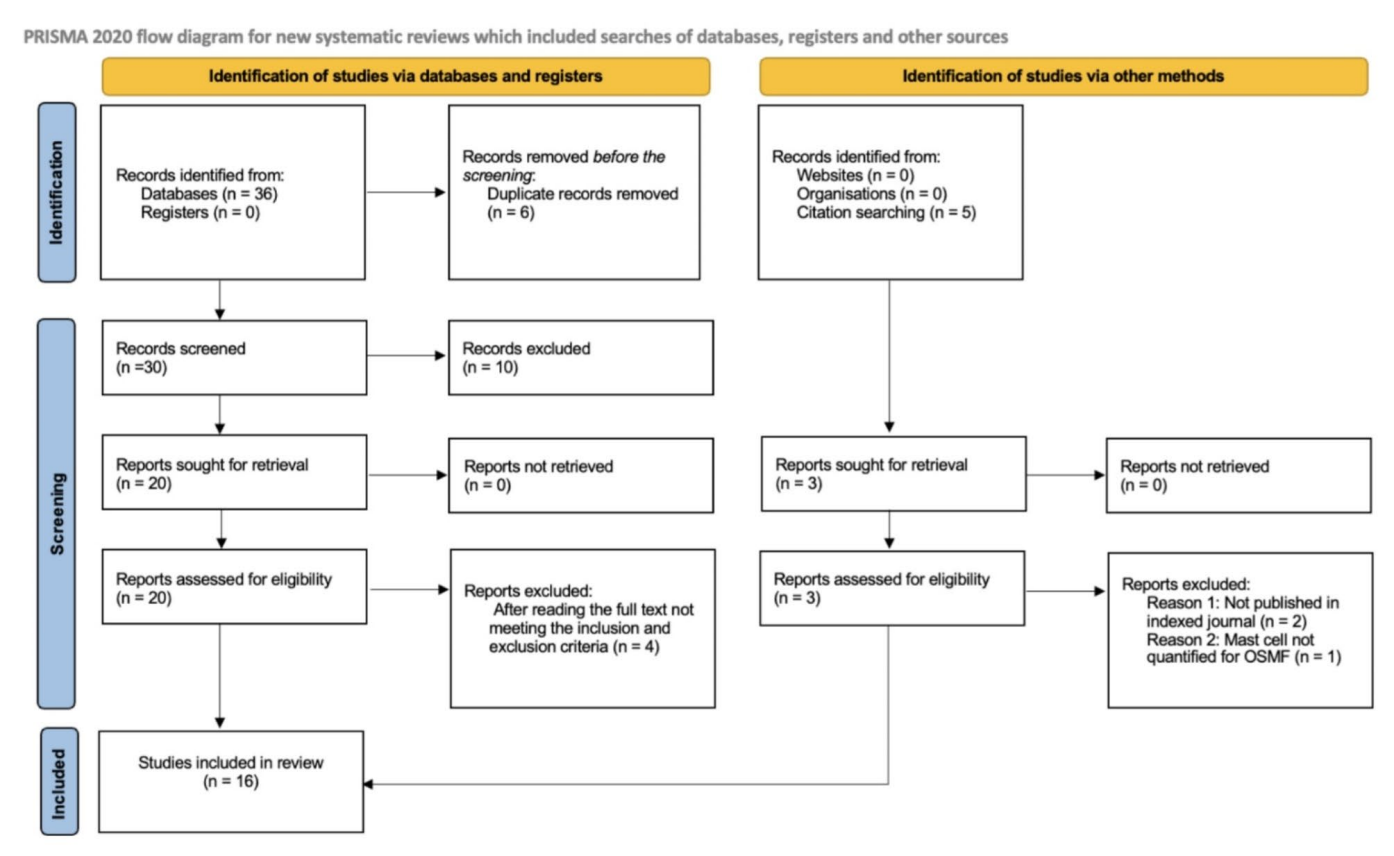
PRISMA flowchart showing study selection for the systematic review

The studies selected were based on the selection criteria and research questions. A meticulous search in PubMed, Scopus, EMBASE, Web of Science, and Google Scholar yielded 36 articles, which we scrutinized. Along with this 5 articles were handpicked from the citations of the studies. Eventually, all 5 articles were not suitable for inclusion. We excluded 6 articles as they were duplicated. Ten articles were excluded after initially reading all the abstracts. Of the remaining 20 articles, 4 articles were excluded after reading the full text based on the inclusion and exclusion criteria. The review included 16 articles eventually to be utilized for the analysis. The selection process is presented as a flowchart as per PRISMA guidelines (Fig. 1) [12].

### Study characteristics

The studies included in this review were based on the analysis of MC count between OSMF and normal mucosa. The study characteristics are summarized in Table 1. Seven authors of the retrieved articles compared the role of MCs with other OPMDs, and OSCC [15, 17, 18, 21, 23–25].

In the present review, we observed MC count and/or density to be measured by all the16 of the included studies. Additionally, microvessel density was studied by Sabrinath B and Seema Gupta et al., [16, 29]. Pereira T et al., and Kumar L.B. et al., assessed the role of intact and degranulated MCs [24, 26]. MC tryptase and chymase were exclusively observed and reported by Yadav A. et al., [21]. A study by Telagi N observed typical, atypical, and granular MCs [21]. Parameters other than MCs, such as lipid profile, eosinophils, and capillary density, were given by Husain B, Pereira T et al., and Nitheash P, respectively [22, 24, 25].

Seven of the sixteen studies were based on the OSMF classification developed by Sirsat SM [14, 16, 18, 19, 27, 29]. Only one study by Kumar LB et al. used both clinical (Kumar KK et al., 2007) and histopathological classification (Utsunomiya et al., 2005) [30, 31]. Khatri MJ adopted the Lai DR classification (1995) [20, 32]. The remaining six studies did not have a detailed classification used in their studies [17, 23–25, 28]. The studies by Yadav (A) et al., and Husain (B) indigenously developed their classifications to report their studies; the former used a clinical classification, whereas the latter author used an indigenous histopathological classification [21, 22].

Thirteen out of the sixteen studies reported using toluidine blue staining for the identification and quantification of MCs. This is due to toluidine blue staining being efficient enough in comparison with IHC, which would require an expensive setup. Five studies used the IHC technique to report on MC count [16, 20, 21, 28, 29].

There was a variation in the MC counting method as assessed by the authors. Five studies assessed using the step ladder fashion on the oculomotor grid [15, 18, 23, 27, 29]. Sirsat SM and Chavan S mentioned counting MCs in their studies from the right upper edge of the oculomotor grid [14, 19]. Yadav A et al., Telagi N, and Gupta S et al. found an average MC count of five fields in the oculomotor grid [21, 23, 29]. Three studies used specific software to evaluate MC count: Khatri MJ used Leica Q Win software, Kumar LB et al. reported using Image J software and Nitheash P utilized Image Prog Res Capture 2.8.8 version software [20, 26, 27]. The remaining authors have not specified counting software or methods [16, 17, 22].

### Study risk of bias assessment

According to the results obtained using the Modified New Castle Ottawa Scale, all the studies scored in the good category (Score 6–9). (Table 2).

**Table 1:** Study characteristics and PICO of the retrieved articles

#	Study	Age	M: F	Classification	MC counting technique	Parameters Observed	Population	Intervention	Control	Primary Outcome	MC count
1	Sirsat SM and Pindborg JJ 1967 [14]	Not specified	Not specified	Sirsat and Pindborg 1966	Right upper edge of occulometer grid.	MC count	80 OSMF	1% Toluidine blue	7 normal buccal mucosa	The MC count in the very early, moderately advanced cases was similar or less than in the normal mucosa.	Case: Very early: 2 Early: 37 Moderately advanced: 31 Advanced: 12 Control: 7
2	Ankle MR, 2007 [15]	Not specified	Not specified	Not specified, Grade I, II, III	Step ladder technique	MC count and density	5 OSMF, 5 oral leukoplakia, 5 lichen planus and 5 OSCC	1% Toluidine blue	5 adult patients undergoing extraction for orthodontic treatment	MC count was highest in oral lichen planus, followed by oral leukoplakia, OSCC, OSMF and the least in normal mucosa	Case: 48.25 Control: 25.50
3	Sabarinath B, 2011 [16]	Not specified	Not specified	Sirsat and Pindborg 1966	Not specified	MCD. MVD, intact and degranulated MC	40 OSMF	Immunohistochemical reagents (anti-Mc tryptase for MCs and anti-factor VIII related von Willebrand factor for endothelial cells)	10 Normal buccal mucosa without any habit	A significant increase in MC density (MCD) and microvascular density (MVD) among OSMF cases. As MCD increases there is an exponential increase in MVD. Intact MCs increased progressively from normal mucosa to moderately advanced OSMF. Degranulated mase cells was higher in very early and early grades of OSMF and lesser in moderately advanced OSMF	Case: Very Early: 223.11 Early: 238.57 Moderately advanced: 201.71 Control: 125.6
4	Kinra M, 2012 [17]	18–55 Mean 27.5	4:1	Not classified	Not specified	MC count and density	10 of OL, OSMF, OLP, OSCC	1% Toluidine blue	10 Control from extraction of third molar	MC count was highest in OSCC, followed by oral leukoplakia, oral lichen planus and OSMF with the least in normal mucosa.	Case: 19.25 ± 4.11 Control: 12.56 ± 4.18
5	Pujari R, 2013 [18]	Not specified	Not specified	Sirsat and Pindborg (1967)	Step ladder technique	MC count and density	25 cases of OSMF, 10 OSCC	1% toluidine blue staining	10 normal buccal mucosa in patients without having any habits	MC count was highest in OSCC, followed by late grade of OSMF and early grade of OSMF with the least in normal mucosa.	Case: 53.25 ± 18.48 Control: 25.00 ±18.488
6	Chavan S, Deshmukh RS, 2013 [19]	OSMF: 15–50 years	Not specified	Sirsat and Pindborg (1967) and Bhatt & Dholakia (1971)	Right upper edge of occulometer grid.	MC density	30 OSMF (20 from pune and 10 from Belgaum)	1% toluidine blue	10 normal buccal mucosa	MC count was highest in Grade III followed by Grade II and same in Grade I and IV. Significant difference between number of MCs of normal mucosa.	Case: 5.80 ± 1.52 Control: 14.90 ± 3.87
7	Khatri MJ, 2013 [20]	15–55 years	15: 2	Lai et al. (1995)	Average of 5 fields, Leica Qwin V3 software	MC count, comparison of MC count between Toluidine blue and c-Kit	30 OSMF	1% toluidine blue and c-Kit	10 controls without any habit	The MC count was higher in OSMF than normal mucosa. The MC density analyzed using toluidine blue and c-kit concluded a significant difference among the stages of OSMF. The mean number of MCs obtained using c-kit was found to be more than that obtained using toluidine blue in various stages of OSMF.	Toluidine blue: Case: 3.16 ± 5.50 Control: 2.18 ± 3.57 c-kit: Case: 8.95 ±1.89 Control: 4.26 ± 3.57
8	Yadav A et al., 2014 [21]	OSMF: 33.9 years	13: 7	Staging based on mouth opening	Average of 5 fields	MC Tyrptase and Chymase	20 OSMF 10 OSCC	Primary monoclonal antibodies used for MC were anti-MC tryptase (Dako) and anti-MC chymase (Abcam).	10 age and sex match healthy volunteers without habits	Subepithelial zone of Stage 1 and 2 while deep zone of Stage 3 and 4 OSMF demonstrated increased tryptase positive MCs.	Case: Subepithelial: 8.10 Deep: 5.68 Control: Subepithelial: 11.68 Deep: 6.52
9	Husain B, 2014 [22]	OSMF: 29.13 ± 1.88 Control: 28.60 ± 1.82	OSMF: 35: 5 Control: 8:2	Gupta PC 2007	Not specified	MC Count, Lipid Profile	40 OMSF	Toluidine blue	10 controls	The MC count is highest in the early grades of OSMF and decreasing with advanced OSMF. MC count was higher in OSMF than normal mucosa. The serum lipid profile decreases in OMSF patients.	Case: 22.50 ± 0.91 Control: 1.40 ± 0.45
10	Telagi N, 2015 [23]	Not specified	Not specified	No classification	Average of 5 zones	Typical, atypical and granular MC	30 Oral epithelial dysplasia, 30 OSMF, 30 OSCC.	Toluidine blue	30 healthy control	The cases with mild, moderate and severe inflammation showed increased number of typical (TMCs), atypical (AMCs) and granular MCs (GMCs), respectively.	Case: 4.90 ± 0.80 Control: 1.40 ± 0.40
11	Pereira T et al., 2018 [24]	OSMF: 20–45 years	19:6 12:1	No classification	Step ladder technique	Intact and degranulated MCs, total eosinophils	25 OPMD of 13 OSMF and 12 leukoplakia 25 OSCC	Toluidine blue	10 Normal tissue control	The mean number of MC and eosinophils was more in OSCC when compared to OPMD (OSMF and Oral Leukoplakia). Intact MCs were more in number in OPMD than in OSCC, and the mean number of degranulated MC was more in OSCC than in OPMD.	Case: 4.8 ± 1.5 Control: 0.4 ± 0.7
12	Dutta J, 2019 [25]	Not specified	Not specified	No classification	Step ladder technique	MC count	5 OSMF 5 oral leukoplakia 5 OSCC	Toluidine blue	2 normal mucosa	OSMF showed highest MC count followed by Oral Leukoplakia, OSCC and normal mucosa.	Case: 33.8 Control: 7
13	Kumar LB et al., 2020 [26]	Case: 35.0 ± 14.1 Control: 37.0 ± 9.9	4:1.	Kumar KK 2007 (Staging) Utsunomiya 2005 (Histopathology)	Image J software	Intact, degranulated and total mast cell. Clinical correlation with burning sensation.	30 OSMF	Toluidine blue	10 healthy individuals	Degranulated MCs were found to have a significant influence in mild to moderate levels of burning sensation among OSMF patients. Role of degranulated MCs were also found to be significant in various clinical stages of OSMF.	Case: 24.70 ± 21.20 Control: 4.20 ± 3.20
14	Nitheash P, 2021 [27]	Grade 1: 20–42 years Grade 2: 35–52 years Grade 3: 45–62 years.	57 Males and 18 Females	Pindborg JJ and Sirsat SM (1966)	Image Prog Res Capture 2.8.8 version software	MC density, epithelial thickness, capillary density, luminal diameter and circumference	75 OSMF	Toluidine blue	10 normal buccal mucosa	MC density was highest in grade 1 OSMF followed by grade 3 and then grade 2. Morphometrical analysis of epithelial thickness and connective tissue changes concluded a varying degree of alterations in different grades of OSMF as the severity of the disease increases.	Case: 6.10 ± 1.14 Control: 7.50 ± 1.30
15	Ashish Shreshta, 2021 [28]	Not specified	67 males and 33 females	No classification	Random Non-overlapping five fields	Mast cell count	15 OSMF 15 lichen planus 15 oral epithelial dysplasia	Toluidine blue and Alcian blue safranin	10 normal mucosa	MC count was highest in epithelial dysplasia, followed by lichen planus, oral submucous fibrosis and OSCC. MC count was higher in OPMD and OSCC than normal mucosa.	Toluidine blue: Case: 7.60 ± 3.28 Control: 4.88± 1.80 Alcian blue safranin: Case: 7.05 ± 2.31 Control: 5.60 ± 1.94
16	Gupta S et al., 2023 [29]	32.8 ± 8.5 years OSMF 29.4 ± 9.2 years Control	4:1	Sirsat and Pindborg (1967)	Average of 5 fields	MC density, Blood vessel mean.	40 OSMF	CD117	10 cases of normal buccal mucosa from age- and sex-match subjects with no habits	The MC density and the number of blood vessels were progressively reduced in OSMF as the grade advanced compared to healthy controls. The advanced cases of OSMF had keratinized epithelium with atrophic changes and moderate to advanced fibrosis of stroma with the involvement of underlying muscles.	Case: 8.12 ± 3.45 Control: 5.37 ± 0.53

**Table 2:** Modified Newcastle Ottawa Scale for analyzing risk of bias [13]

	Author (Year)	Selection				Comparability	Exposure			Total Score For 10 (Quality)
		Case Definition	Representativeness of the cases	Selection of controls	Definition of control	Comparability	Ascertainment of exposure	Ascertain-ment for cases and controls	Non-Response rate	
1	Sirsat SM and Pindborg JJ 1967 [14]	1	1	0	1	1	0	1	1	6 (Good)
2	Ankle MR 2007 [15]	1	1	1	1	1	0	1	1	7 (Good)
3	Sabarinath B 2011 [16]	1	1	1	1	1	1	1	1	8 (Good)
4	Kinra M, 2012 [17]	1	1	1	1	2	0	1	1	8 (Good)
5	Pujari R, 2013 [18]	1	1	1	1	1	0	1	1	7 (Good)
6	Chavan S, Deshmukh RS, 2013 [19]	1	1	0	1	2	1	0	1	7 (Good)
7	Khatri MJ, 2013 [20]	1	1	1	1	2	1	1	1	9 (Good)
8	Yadav A et al., 2014 [21]	1	1	1	1	2	1	1	1	9 (Good)
9	Husain B, 2014 [22]	1	1	1	1	2	1	1	1	9 (Good)
10	Telagi N, 2015 [23]	1	1	1	1	1	0	1	1	7 (Good)
11	Pereira T et al., 2018 [24]	1	1	1	1	2	0	1	1	8 (Good)
12	Dutta J, 2019 [25]	1	1	1	1	1	0	1	1	7 (Good)
13	Kumar LB et al., 2020 [26]	1	1	1	1	2	1	1	1	9 (Good)
14	Nitheash P, 2021 [27]	1	1	1	1	2	0	1	1	8 (Good)
15	Ashish Shreshta, 2021 [28]	1	1	1	1	2	1	1	1	9 (Good)
16	Gupta S et al., 2023 [29]	1	1	1	1	2	0	1	1	8 (Good)

**Fig. 2:**
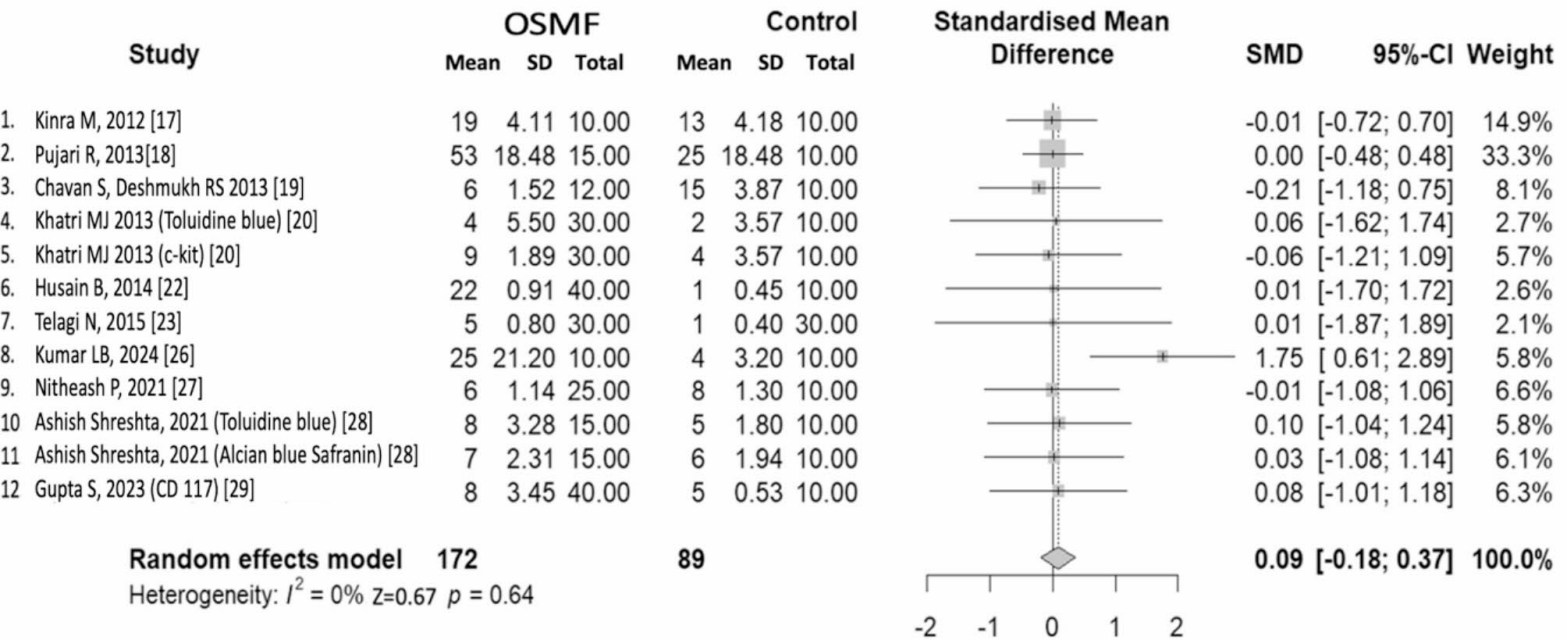
Forest Plot of pooled effect size depicting differences in MC count between OSMF and Control

The meta-analysis was performed with R studio software (Version: 4.4.0, Year: 2024, Company: The R foundation for statistical computing). The standard mean difference was analyzed for comparison of MC counts between the studies. From the forest plot, we can presume that the effect size of most of the studies was high. Most studies have high confidence intervals which can indicate less precision of the sample estimates. There is a high level of homogeneity in results as the i^2^ percentage is zero. All this is due to the sampling errors observed in these studies. The case-to-control ratio in all the studies was less than 1:1. The pooled effect size hence does not conclusively indicate the role of MC as its confidence interval lies on either side of the line of no effect. Studies by Kinra M, Pujari R, Chavan S, Khatri MJ (c-kit), Husain B, Telagi N and Nitheash P overlap with lines of no effect [17–20, 22, 23, 27]. Studies by Khatri MJ (c-kit), Husain B and Telagi N indicate very high confidence intervals [20, 22, 23]. Hence not able to correlate the role of MC with OSMF. Studies by Khatri MJ (Toluidine blue), Kumar LB, Ashish Shrestha and Gupta S show the correlation of MCs with OSMF [20, 26, 28, 29] (Fig. 2).

## Discussion

Oral Submucous fibrosis is a potentially malignant disorder as defined by Pindborg (1966) as an insidious chronic disease affecting any part of the oral cavity and sometimes the pharynx. It is associated with a juxta-epithelial inflammatory reaction followed by fibroelastic changes in the lamina propria layer, along with epithelial atrophy which leads to rigidity of the oral mucosa proceeding to trismus and difficulty in mouth opening [5]. Globally, the malignant transformation rate of OSMF is estimated to be 4.2% (95% CI 2.7–5.6%) [33].

Mast cells have been extensively studied both in physiological and pathological states and the progenitors of MCs when activated tend to co-habit in the sub-epithelial region. The role of MCs has been established in inflammatory conditions such as gingivitis, pulpitis, periapical inflammation, and oral lichen planus [34, 35].

Sirsat and Pindborg gave the first classification of OSMF in 1966 and subsequently in 1967. The elaboration of histopathological grades from three to four is completely developed based on the histopathology of OSMF [5, 14]. Kumar LB et al., utilized the classification to establish the correlation between burning sensation in OSMF and MC count [26]. The histopathological classification given by Sirsat and Pindborg varies from the classification given by Utsunomiya (who simplified the classification given by Sirsat and Pindborg into 3 grades by combining), and the Lai classification is based on mouth opening and is clinically based [5, 31, 32].

The present systematic review adds to previous literature by including studies reported recently as well as attempts to critically examine and bring about an understanding of various aspects of the role of MC count (intact, degranulated, total, atypical, typical, granular, tryptase, and chymase).

Bhat and Dholakia were the first to report that degranulated MCs are associated with an itching sensation in the oral mucosa due to histamine release in their study of OSMF [36]. Kumar LB et al., improved on a similar observation by assessing the intact and degranulated MC role in the burning sensation of the mouth based on their case-control study [26]. Chavan S, Kumar LB et al., and Gupta S et al., used more than one observer to overcome intra-observer bias and ensure reliability [19, 26, 29].

Literature has clearly stated that immunohistochemistry can identify MCs better than toluidine blue [21, 28, 37]. Khatri MJ reported that c-kit is a more reliable technique to assess MC density in OSMF [20]. Ashish Shreshta concluded that the MC count for the Alcian blue-safranin stain was higher than the toluidine blue stain [28]. Yadav A et al., reported mast cell density using anti-MC tryptase (Dako) and anti-MC chymase (Abcam) [21]. Yadav A. et al., solely reported MCs to contain predominantly tryptase, followed by some containing tryptase and chymase; rarely chymase-positive MCs are observed in OSMF; They also observed a decreasing trend in tryptase positivity as the stage of OSMF progressed. The role of tryptase and chymase within MCs, which could serve as potential pathological indicators of OSMF, must be further studied and validated [21].

All the studies concluded that the MC count was increased in OSMF when compared to normal mucosa. In studies conducted by Sirsat SM, Sabarinath B, Khatri MJ, Chavan S, Husain B, and Gupta S et al., the MC count decreased in advanced cases of OSMF [14, 16, 19, 20, 22, 29]. However, there were conflicting reports by Pujari R. and Kumar LB et al., who reported a gradual increase in MCs as the stages of OSMF advanced [18, 26]. Studies reported among other OPMDs suggested that MCs were higher-order oral leukoplakia than OSMF. MCs were higher in OSCC than OSMF in studies by Ankle MR, Kinra M, Pujari R, Yadav A, et al., Telagi N, and Pereira T et al., [15, 17, 18, 21, 23, 24]. However, there were conflicting results from Dutta J [25]. 

**Fig. 3:**
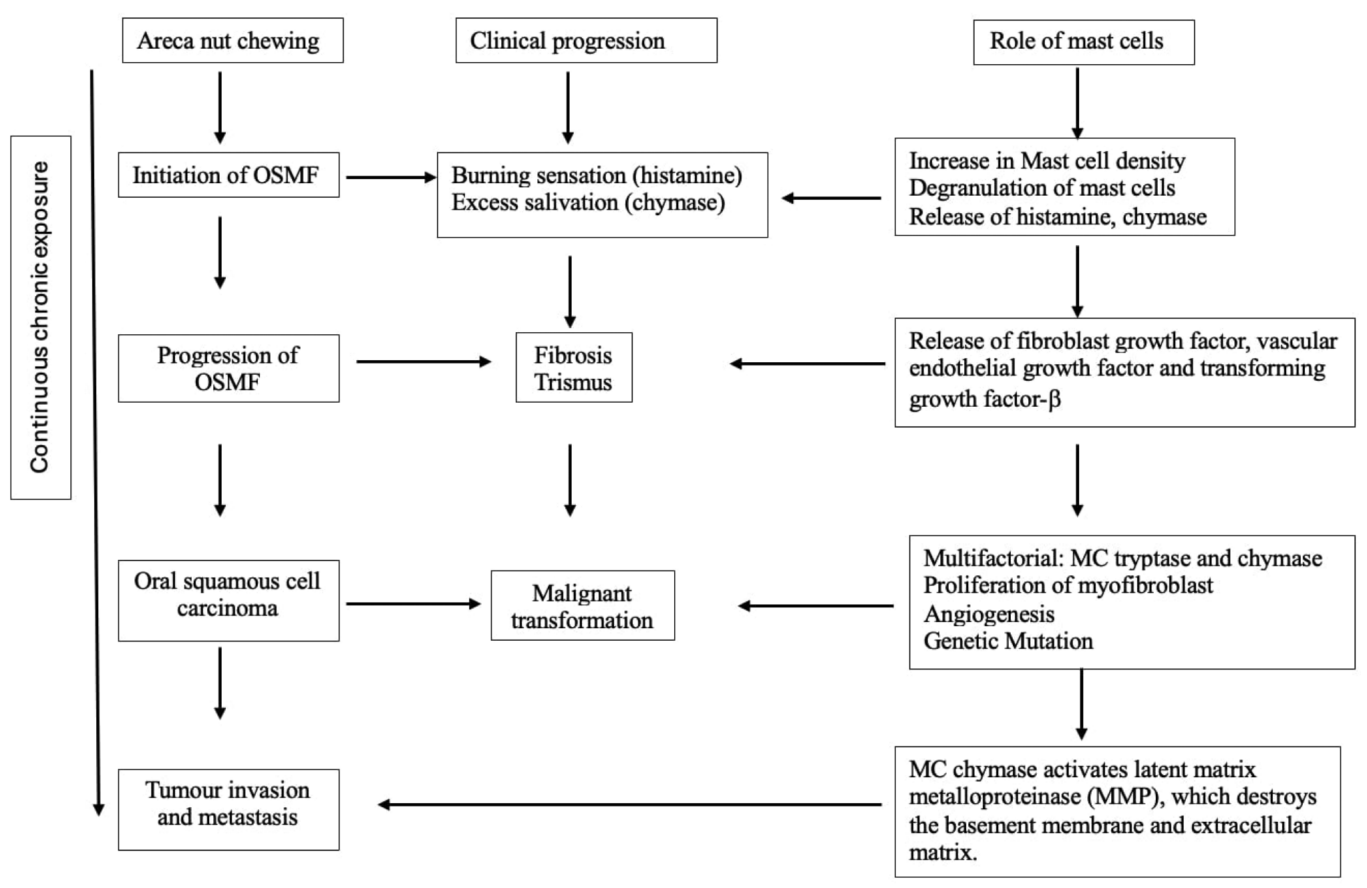
Proposed flowchart for the role of mast cells in OSMF

Histamine is released upon degranulation of MC and contributes to edema in the submucosa in the early stages of OSMF. Eosinophilic chemotactic factor (ECF) released from the MCs contributes to the eosinophils which can be a part of the inflammatory cell infiltrate seen in the early stages of OSMF [38, 39]. These findings were also consistent with Pereira T et al., [24] The release of interleukin − 1 from the MC may increase the fibroblastic response and MC tryptase. This can increase the production of type 1 collagen and fibronectin which in turn increases fibrosis [40]. Yadav A et al., also had consistent findings that MC tryptase was the main mediator for fibroblast proliferation, both in the early and advanced stages of OSMF [21]. MC tryptase was higher than MC chymase in the subepithelial and deeper zones. MC chymase has been known to activate latent matrix metalloproteinase (MMP), which destroys the basement membrane and extracellular matrix. This is crucial for tumor invasion and metastasis. Also, they observed a significant increase in MC tryptase and chymase in OSCC than in OSMF. They also concluded that MC tryptase and chymase contributed to the malignant transformation of the overlying epithelium [21]. The mean degranulated MC density is higher than the intact MC density in OSCC. This suggests that MC granules have a role in tumour progression [23]. However, this contradicts the findings observed by Sabarinath B and Kumar LB et al., [16, 26]. Based on the available literature the proposed role of mast cells in OSMF is given in Fig. 3.

In the present study, Modified New Castle Ottawa was used to assess the risk of bias [11]. One of the similar systematic reviews on the topic used SIGN grading for the same [37]. The authors felt SIGN grading to be inappropriate as not much emphasis is given to clinical considerations while studying or analyzing this topic as this is a histopathological analysis and not an etiological analysis [35]. Though studies relevant to this topic mention the study design as case-control, hardly matching for cases is carried out while a selection of controls. None of the studies have reported more than a 1:1 case-to-control ratio nor are confounders analyzed in these studies. It is purely a histopathological analysis. Based on the meta-analysis there is vast heterogeneity in sampling. There needs to be a 1:2 or more case-to-control ratio for analyzing the role of MC in OSMF. By having more controls, the matching-related bias will be reduced. It also helps in establishing a better association with etiology.

The strength of this systematic review is that it has analyzed the long-standing debate on the role of MC in OSMF and its progression. In a nutshell, our review could confirm the following points. The role of MC count is currently limited to the early stages of OPMDs. There is a lack of uniformity in the methodology in the literature. The sample size in all the studies in this review was insufficient and is mostly confined to one geographical area (India). These findings will help researchers to bring in more methodological rigor while researching this topic. The limitations of the systematic review may include that the authors might have missed out on certain repositories and articles from other sources which can be overcome if future studies are undertaken are consistent with the present topic. The reporting of MC count is observed to be heterogeneous about the type of MC observed, method of staining, method of calculating MCs, MC count (density and count), classification of OSMF (clinical, histopathological, functional, or both), and counting of MC in the subepithelial zone or deep zone. There are variations also in the parameters observed such as the associated symptom (burning mouth, thickened oral mucosa, restricted mouth opening or movement of the tongue, and difficulty in deglutition). The comparison with other conditions is also variable such as either normal mucosa, specific OPMD, or OSCC. The research is carried out predominantly in the South Asian Region, where the prevalence and incidence of OSMF are higher and research is carried out, observing low grant facilities, and most of the studies reporting are self-funded.

## Conclusion

The present systematic review would like to conclude by stating that MC count has a definitive role in the pathogenesis of OSMF. However, there is an observation of heterogeneity regarding the methodology being used to analyze the role of MCs. There needs to be consensus on the rigor of the methodology and once the methodological consensus is in place to analyze MC the validity of studies would increase, which would further contribute to the establishment of causation. A multi-center study would add more understanding to the role of MCs in OSMF, not just limited to one geographical region but throughout the South Asian region. With thorough knowledge of the role of mast cells in OSMF, future studies can be aimed at drug trials using mast cell stabilizers for management.

## Data Availability

The datasets generated and/or analyzed during the current study are available in the following link. Additional details will be available from the corresponding author on reasonable requesthttps://tinyurl.com/supplemental-file.

## Abbreviations

OSMF: Oral Submucous Fibrosis
OSCC: Oral Squamous Cell Carcinoma
OPMD: Oral Potentially Malignant Disorders
MC: Mast Cell
MCC: Mast Cell Count
MCD: Mast Cell Density
MVD: Mean Vascular Density
SMD: Standard Mean Deviation
TNF: Tumour Necrosis Factor
MMP: Matrix Metalloproteinases
